# Understanding Early Treatment Response in Brief CBT for Nonunderweight Eating Disorders: A Mixed Methods Study

**DOI:** 10.1002/eat.24350

**Published:** 2024-12-13

**Authors:** Dana Gatley, Verity Millar‐Sarahs, Amy Brown, Cat Papastavrou Brooks, Faith Matcham

**Affiliations:** ^1^ School of Psychology University of Sussex Brighton UK; ^2^ Sussex Partnership Innovation and Research in Eating Disorders (SPIRED) Clinic Sussex Partnership Foundation Trust Sussex UK; ^3^ Sussex Eating Disorder Service Sussex Partnership Foundation Trust Sussex UK; ^4^ Population Health Sciences University of Bristol UK

**Keywords:** 10‐session cognitive behavioral therapy, atypical anorexia nervosa, binge‐eating disorder, bulimia nervosa, CBT‐T, early change, nonunderweight eating disorders, OSFED, predictors

## Abstract

**Objective:**

Early change in eating disorder psychopathology is the most robust predictor of treatment outcomes in eating disorders. However, little is known about what predicts early change. Using mixed‐methodology, this study explored predictors of early change in the first four sessions of 10‐session cognitive behavioral therapy (CBT‐T) for nonunderweight eating disorders.

**Method:**

Phase 1: interviews were conducted to explore CBT‐T clinicians' perspectives on predictors of early change. Phase 2: robust multiple regressions were undertaken to examine whether any of five variables identified during interviews—diagnosis, wait time, therapeutic alliance, depression, and anxiety—were associated with early change in eating disorder psychopathology. Data were derived from outcome measures for service users (*n* = 107) receiving CBT‐T in a community eating disorder service.

**Results:**

Phase 1: eight themes were identified: attitudes to making change, diagnosis, external mitigating circumstances, therapeutic alliance, therapist confidence, pretreatment variables, CBT‐T format, and therapeutic suitability. Phase 2: no significant associations were found between the five predictor variables (diagnosis type, wait time, baseline depression, baseline anxiety, and therapeutic alliance) and early change in EDE‐Q scores. These results have been certified as computationally reproducible by an independent statistician.

**Discussion:**

Qualitative findings identified several potential predictors of early change in eating disorder psychopathology in CBT‐T, however, quantitative data contradicted qualitative findings, finding no significant association for any of the tested variables. Further research is required to clarify theses conflicting findings and to quantitatively explore the additional predictors highlighted during qualitative analysis.

**Summary**

- This study underscores 10‐session cognitive behavioral therapy's efficacy in promoting early change in nonunderweight adults with eating disorders and provides valuable insights into clinicians' perceptions of the factors influencing early change.
- However, quantitative analysis contradicted qualitative findings, finding no significant predictors of early change.
- These findings highlight the need for ongoing research to guide treatment suitability and improve treatment outcomes for individuals with nonunderweight eating disorders.

## 1 Introduction

Nonunderweight eating disorders (EDs), including binge‐eating disorder (BED), bulimia nervosa (BN), and atypical‐anorexia nervosa, represent over half of ED outpatients (Mancuso et al. 2015; Vo et al. 2017). Thought to affect 1%–10% of women during their lifetime (Galmiche et al. 2019), nonunderweight EDs are associated with adverse outcomes including increased psychological and physical morbidity (Keski‐Rahkonen et al. 2009), increased healthcare costs (Jenkins 2022), and increased mortality and risk of suicide (Keski‐Rahkonen and Mustelin 2016).

At present, the recommended treatment for nonunderweight EDs is up to 20 sessions of ED‐focused cognitive behavioral therapy (CBT‐ED; National Institute for Health and Care Excellence 2017). However, longer psychological treatments are not associated with superior outcomes for EDs (Radunz et al. 2020), and waitlists for treatment are long (Austin et al. 2021). Hence, there is pressure for shorter, more cost‐effective interventions, particularly as earlier access can improve treatment outcomes (Ambwani et al. 2020).

Waller et al. (2019) developed a shorter, 10‐session cognitive behavioral therapy (CBT‐T) for nonunderweight EDs. CBT‐T is manualized, so can be delivered by both qualified and unqualified clinicians, further increasing access to therapy. The protocol includes self‐monitoring of food intake, weekly weighing, psychoeducation, behavioral and nutritional change, exposure, behavioral experiments, cognitive restructuring, body image work, and relapse prevention (Paphiti and Newman 2023).

CBT‐T case series studies indicate significant reductions in ED psychopathology (Moore, Hinde, and Waller 2021; Pellizzer, Waller, and Wade 2019; Waller et al. 2018) and patients appear to find it acceptable (Hoskins et al. 2019). Meta‐analyses show CBT‐T produces comparable outcomes to CBT‐ED in half the time (Keegan, Waller, and Wade 2022; Paphiti and Newman 2023). Although CBT‐T has been introduced across NHS services, only one randomized control trial has been conducted (Wade, Ghan, and Waller 2021), making continued research imperative to understand its effectiveness, mechanisms of change and suitability for different patient subgroups.

### 1.1 Importance of Predicting Early Change

CBT‐T emphasizes the importance of making behavioral change early in treatment as this has been shown to be the most robust predictor of ED treatment outcomes (Chang, Delgadillo, and Waller 2021; Vall and Wade 2015). After review in session 4, treatment is extended to the full 10 sessions only for clients who have made changes; for others, additional sessions are unlikely to be beneficial (Waller et al. 2019), and they are encouraged to return to treatment in the future when they are more ready to engage.

Approximately 50% of patients make substantial early changes in psychological therapies but the mechanisms behind this remain poorly understood (Chang, Delgadillo, and Waller 2021). The value of research exploring and identifying mechanisms of change is emphasized in the new MRC complex intervention framework (Skivington et al. 2021). In CBT‐T, gaining an understanding of factors that influence early change would bring important benefits for services and service users. First, it would facilitate clinical decision‐making around treatment allocation, ensuring resources are used effectively (Lutz et al. 2014) and benefitting clients, particularly as therapy requires considerable practical and time commitment. Second, it may help decrease the likelihood of clients suffering adverse effects during treatment: approximately 5%–10% of patients deteriorate in psychological therapy (Lambert and Ogles 2004), with 5% reporting lasting negative effects (Crawford et al. 2016). Experiencing poor outcomes in therapy causes stress and reduces self‐esteem and self‐efficacy (Rozental et al. 2016). Third, if clinicians understand what predicts poorer outcomes, this could not only inform their decisions about treatment suitability (Castonguay et al. 2010), it could also allow factors which limit a client's ability to make early change to be targeted through interventions or adjustments prior to starting treatment, so they enter CBT‐T in a better position to succeed.

There has been no research to date into predictors of early change in CBT‐T. Research in other psychological therapies for EDs is limited and has produced contradictory findings. In anorexia nervosa (AN), early weight gain in therapy has been associated with lower ED symptomology at onset and greater optimism toward treatment (Hughes et al. 2019), as well as with absence of comorbidities (Le Grange et al. 2014). However, these findings are not well replicated. Recent research in patients with BED found early change was not predicted by sociodemographic or clinical variables, including comorbidity, but was more likely in patients with more positive attitudes toward treatment (Yurkow, Ivezaj, and Grilo 2023).

Research into other mental health problems suggests early symptom change can be influenced by therapist factors, such as therapist adherence in treatment for depression (Strunk, Brotman, and DeRubeis 2010), and alliance negotiation in treatment for emotional disorders (Manubens et al. 2023). The inconclusive nature of findings solidifies the need for further studies exploring predictors of early change, with service‐user characteristics including readiness to change (Lutz et al. 2014) and ability to tolerate anxiety while making changes (Chang, Delgadillo, and Waller 2021) being raised as valuable research areas.

The present study, run in collaboration with the Sussex Eating Disorder Service (SEDS), an NHS adult community eating disorder service, aimed to address this gap in the literature by exploring predictors of early change in ED psychopathology in the first four sessions of CBT‐T. An exploratory sequential mixed‐methods design was used (Creswell, Gutmann, and Hanson 2003) to allow for more comprehensive understanding of this novel topic and increase confidence in the findings (Creswell and Clark 2007). In the first phase, qualitative interviews with SEDS clinicians who deliver CBT‐T were conducted to explore their perspectives on factors influencing early change in ED psychopathology. Themes from these interviews informed the second, quantitative phase, designed to test predictors of early change using routinely collected SEDS CBT‐T outcome measures.

## 2 Phase 1: Qualitative Investigation

### 2.1 Method

#### 2.1.1 Participants

SEDS clinicians who deliver CBT‐T were invited for interview via email and anyone expressing an interest was sent a participant information sheet, along with a consent and demographics form for completion. Service users were not interviewed due to pragmatic considerations around the timeline of the project. The clinicians had been trained and received on‐going weekly group supervision from one of the authors of the CBT‐T manual. All eight invited clinicians consented and completed interviews, lasting 30–62 min (*M* = 46.18, SD = 12.03), and received a £20 Love2Shop voucher. Two participants were interviewed twice as the original audio recordings became corrupted. Due to the high specificity of the sample, broad study aim and high quality of the dialogue, the sample was determined to have sufficient information power (Malterud, Siersma, and Guassora 2016). Table 1 describes participant characteristics.

**TABLE 1:** Description of interview participant sample in Phase 1.

	Participants
Categorical	*n*
Gender	
Cisgender female	7
Cisgender male	1
Ethnicity	
White British	6
Asian British	1
Mixed ethnicity	1
Highest educational level	
Undergraduate	4
Postgraduate	4
Job title	
Assistant psychologist	5
Senior assistant psychologist	1
Eating disorder practitioner	2

Continuous	*M* (SD)	Min–Max
Age (years)	26.13 (3.52)	22–32
Time working in ED services (months)	21.50 (11.60)	6–36

Abbreviations: *M* = Mean; Max = maximum; Min = minimum; SD = standard deviation.

#### 2.1.2 Materials

A semi‐structured interview topic guide (Data S1) was created by DG and reviewed by the Sussex Partnership Innovation and Research in Eating Disorders (SPIRED) clinic and a member of SEDS' Lived Experience Advisory Panel. The guide was designed to explore clinicians' experiences with CBT‐T, centering on their perceptions of the factors influencing service‐user outcomes in the first 4 weeks of treatment.

#### 2.1.3 Procedure

Audio‐recorded interviews were conducted via Zoom then transcribed verbatim by DG and VM‐S. Transcripts were anonymized, with all potentially identifiable information removed, and allocated PINs stored in a password‐protected spreadsheet on secure servers.

#### 2.1.4 Data Analysis

Transcripts were analyzed using NVivo software (QSR International 2023) and reflective thematic analysis (TA), following the six phases outlined by Braun and Clarke (2006). An inductive “bottom‐up” TA approach was utilized to allow greater flexibility. As the first study to explore predictors of early change in CBT‐T, it was important for analysis to be driven by the data rather than by a theoretical framework, allowing identification of unexpected themes. The presence of any diverse cases was also considered in analysis.

Both DG and VM‐S familiarized themselves with the data using both audio recordings and transcripts of the interviews. All transcripts were then coded by DG and a subsection were double‐coded by VM‐S to broaden the range of interpretations of the data. Both coders independently developed code clusters and candidate themes, and then met to discuss coding decisions and initial theme ideas. The candidate themes were then refined and revised by DG in an iterative process, which included revisiting the transcripts and recoding until the code could be finalized. Both researchers were white women with limited prior experience of TA.

At the time of the research, both DG and VM‐S were working within SEDS: this facilitated recruitment of participants (who were familiar with this project and the authors) and supported rapport during interviews. Additionally, as DG and VM‐S were working within SEDS, their analysis potentially accentuated a clinical perspective, as it was shaped by insider awareness of the clinical and administrative backdrop to CBT‐T.

### 2.2 Results

Eight themes were developed from the data. A breakdown of the themes, alongside illustrative quotes, is shown in Table S1.

#### 2.2.1 Service‐User Factors

##### 2.2.1.1 Attitudes to Making Change

Service users' attitudes to making change were considered key for early change. All eight clinicians emphasized the importance of individuals being highly motivated upon entering treatment and ready to make change and “give up” their ED, as no time is spent developing motivation within CBT‐T:

“… the people who are not very attached to their eating disorder and are really motivated and they say, I'm 10 out of 10 motivated. I'm ready to make change right now […] They're not contemplating change. They would do it right now if someone […] just told them what to do […] Those people will do really well.” (Clinician 2)

Clinicians also suggested that service users needed to believe they were able to make changes, as those that do not may be less willing to try. The changes required in CBT‐T are anxiety‐inducing, and clinicians noted greater early change in individuals who understood the rationale and “pushed through” their anxieties to attempt changes, particularly if they immediately “dived‐into” dietary changes, as this meant they saw physical improvements and disproved their fears around weight‐gain more quickly than those making gradual or inconsistent changes. This then helped to reduce the anxiety these behaviors caused and built trust in the treatment process, motivating further change:

“… it's okay to be scared, like of things like weighing yourself, of things like doing food diaries or eating fear foods or eating regularly, but the people that do the best are the people that are scared and kind of can do it anyway because they understand […] the rationale, and they're not too, like resistant to that.” (Clinician 2)

Additionally, as CBT‐T is a “doing therapy” in which service users “get out what they put in,” those who engaged in, committed to, and prioritized treatment were seen to have better early outcomes. Finally, as the first four sessions do not include identity work, making change was perceived to be harder for individuals who viewed their ED as part of their identity, particularly if they had nothing to “replace” it with.

##### 2.2.1.2 Diagnosis‐Related Factors

Clinicians discussed the impact of diagnosis on early change. All eight clinicians felt CBT‐T was highly effective for binge‐eating type diagnoses, and the manual clearly laid out how to treat this group. However, clinicians believed the manual's focus on binge‐eating symptomology made CBT‐T harder to adapt to restrictive presentations:

“I don't think it necessarily works as well for OSFED or atypical AN. I think there's a lot of things in there, even kind of the talk of binging. Some patients will be put through and they're not binging at all, and I think then trying to adapt to sessions can be quite difficult.” (Clinician 1)

This meant clinicians felt CBT‐T was often less effective with this group who could struggle to make changes, unless they started treatment relatively “well” and motivated, perhaps having completed prior treatment or weight restoration. Additionally, three quarters of clinicians felt longer ED durations made early change more challenging, as behaviors were more ingrained; however, others reported experiences contrary to this.

##### 2.2.1.3 External Mitigating Circumstances

This theme captured the way factors external to someone's ED may influence their ability to make early change. Several factors were seen as potential obstacles to early change. First, life events and stressors—both when unexpected, such as job loss, and when routine, such as childcare responsibilities—were seen as barriers to engagement. Clinicians described the need for it to be the “right time,” as CBT‐T requires considerable commitment to be effective. Second, comorbidity was seen to hinder early change. For example, anxiety disorders may make it harder to attempt anxiety‐inducing changes, and depression may create issues around motivation and engagement, as described by clinician 7:

“… if someone, alongside eating disorder is struggling with anxiety […] the idea of making changes in one area, you know, they can voice a lot of thoughts and sort of catastrophisation around the idea, which can hinder their ability to make those changes […] if someone's very low in mood or struggling with depression, then that can also impact motivation and ability to do things […] So actually the idea of eating regularly is difficult, but not necessarily because the eating itself, but actually the doing things …”

Clinicians felt some patients may have made better early change if their comorbidities had been managed prior to starting CBT‐T. Third, half of clinicians described seeing many patients with a trauma history which, if “unprocessed,” may “come to the surface” in CBT‐T. As the treatment is short and present‐focused, clinicians considered it unsuitable for tackling trauma and that this could be difficult to contain, particularly if someone's ED was a coping mechanism for their trauma. Conversely, social support was considered beneficial to making early change, by providing a source of motivation and accountability.

#### 2.2.2 Therapist Factors

##### 2.2.2.1 Significance of the Therapeutic Alliance

While the CBT‐T manual does not emphasize the importance of the therapeutic alliance on a patient's ability to make change, all clinicians expressed a level of disagreement with this stance, arguing that their relationship with service users assisted them in making early change, by helping them feel safe and supported. A strong therapeutic alliance was considered essential in creating the required trust in the treatment and therapist when asking individuals to make difficult changes:

“I think if you don't have that rapport, you know, you don't work on building that therapeutic relationship and […] developing that trust in the person you're working with that actually you've only just met, […] Asking someone to make changes, asking someone to trust you and to sort of believe what you're telling them and to have faith, have trust that if they do these things, it could help […] I think that would just be detrimental if you didn't have that rapport.” (Clinician 7)

Moreover, clinicians felt that therapeutic relationships foster motivation—particularly for patients lacking social support—by helping them feel someone was recognizing their efforts and rooting for them, and by providing hope that change is possible.

##### 2.2.2.2 Therapist Confidence

Clinicians' confidence in their ability to deliver CBT‐T was seen as important in supporting early change. Two clinicians expressed awareness that their own anxieties could limit the extent to which dietary change was “pushed” for, potentially limiting patients' change. For example, Clinician 2 said:

“… the more confident you get with delivering CBT‐10, the more comfortable you are with pushing someone the right amount […], because it's a therapy that's so focused on like making active change, you do have to push people quite a lot and you worry if you're pushing them a bit too much and then they might drop out or run away or not want to do it …”

Training and experience were viewed as key to reducing these anxieties and developing confidence in delivering CBT‐T. In particular, clinicians thought receiving supervision from one of the creators of CBT‐T played a vital role in increasing their confidence around adapting the treatment to better suit patients.

#### 2.2.3 Service and CBT‐T Factors

##### 2.2.3.1 Pretreatment Variables

This theme captured the way service users' experiences, while waiting for treatment may impact their ability to make early change. Long wait times were considered detrimental to early change, as presentation and self‐efficacy could deteriorate while waiting. Clinicians associated longer waits with patients having a poorer idea of what to expect from treatment, meaning they were less “set‐up” for CBT‐T and less ready to make change. Clinician 2 described one experience with this:

“… one of the things she said was “my expectations weren't set up for this. No one told me I was gonna have to eat three meals.” And I think because she'd been on a waiting list for such a long time […] and no one told her after assessment like, this is CBT‐10, you're on the waiting list for this. […] And then suddenly she gets a call from me after […] probably over a year, going, […] we've got a slot for you in treatment. She has no idea what treatment is …”

However, clinicians viewed therapeutic contact while waiting as beneficial; most described providing orientation sessions to help set expectations and prepare people for treatment. Over half suggested more established waiting list contact, such as short motivational or psychoeducation groups, could improve outcomes by preparing service users to make changes.

##### 2.2.3.2 CBT‐T Format

The format of CBT‐T was seen to “work” for some, but not all, patients. Most clinicians reflected that while for some service users, the length and pace of CBT‐T effectively encouraged change, for others “making all the changes was too overwhelming and the pace was too fast” (Clinician 6) and it left them feeling frustrated. Additionally, some responded well to CBT‐T's structure but for others it was seen as being too inflexible, potentially limiting their ability to make change.

##### 2.2.3.3 Therapeutic Suitability

Clinicians felt CBT‐T is “quite a specific treatment for a specific group of people” (Clinician 1) and was often delivered to service users who were not well suited to it. Some felt a clearer understanding of CBT‐T suitability is needed to facilitate allocation. For some patients, there are no available alternative treatments if CBT‐T does not “suit” them and clinicians thought these patients could benefit from development of a longer treatment, focusing more on motivation, identity, and restrictive presentations.

## 3 Phase 2: Quantitative Investigation

### 3.1 Method

Phase 1 highlighted factors, which clinicians believed predicted early change in ED psychopathology. From these, five predictor variables could be explored quantitatively using the available service‐user data: diagnosis type, wait time, baseline depression, baseline anxiety, and therapeutic alliance.

#### 3.1.1 Participants

Participants were adults (aged ≥ 18 years) who received at least one session of CBT‐T within SEDS. To be eligible for CBT‐T, service users must have been diagnosed with an ED at assessment using DSM‐5 criteria (American Psychiatric Association 2013) and have a BMI ≥ 18.5.

#### 3.1.2 Materials

Data were collected as part of routine clinical care within SEDS. Patient records were used to compile data on diagnosis type and wait time, along with demographic data including age, gender, and BMI at treatment onset. Other data came from outcome measures contained within questionnaires, which patients completed prior to each CBT‐T session. The measures included in this project's analysis are described below.

##### 3.1.2.1 Diagnosis Type

ED diagnoses were categorized as either “Binge Eating” or “Restrictive” (Table 2).

**TABLE 2:** Description of the categorization of DSM‐V diagnoses into binge eating or restrictive type groups.

	Diagnosis type	
DSM‐V diagnosis	Binge eating	Restrictive
Bulimia nervosa (BN)	X	
Binge‐eating disorder (BED)	X	
Avoidant/restrictive food intake disorder (ARFID)		X
Other specified feeding or eating disorder (OSFED)		
Bulimia nervosa (OSFED‐BN)	X	
Binge‐eating disorder (OSFED‐BED)	X	
Atypical anorexia nervosa (OSFED‐AAN)		X
Purging disorder (OSFED‐Purging)		X
Unspecified feeding or eating disorder (UFED)		X

##### 3.1.2.2 Wait Time

Number of weeks between referral and treatment onset.

##### 3.1.2.3 Baseline Depression

The patient health questionnaire‐9 (PHQ‐9; Kroenke, Spitzer, and Williams 2001), administered before session 1, is a 9‐item measure of symptoms of depression. PHQ‐9 has good sensitivity, specificity and internal consistency (*α* = 0.86–0.89) (Kroenke et al. 2010).

##### 3.1.2.4 Baseline Anxiety

The generalized anxiety disorder‐7 (GAD‐7; Spitzer et al. 2006), administered prior to session 1, is a 7‐item measure of symptoms of generalized anxiety. GAD‐7 has demonstrated good reliability and internal consistency (*α* = 0.89) (Löwe et al. 2008).

##### 3.1.2.5 Therapeutic Alliance

The Working Alliance Inventory—Short Revised (WAI‐SR; Hatcher and Gillaspy 2006), administered before session 4, is a 12‐item measure of the quality of therapeutic alliance. WAI‐SR is based on the Working Alliance Inventory (WAI; Horvath and Greenberg 1989) and has been shown to correlate with the original WAI and have strong internal consistency (*α* = 0.92) (Hatcher and Gillaspy 2006). WAI‐SR is divided into three dimensions, Goal, Task, and Bond, averaged to create an Overall score.

##### 3.1.2.6 Change in Global ED Psychopathology

The Eating Disorders Examination Questionnaire (EDE‐Q; Fairburn and Beglin 2011) is a 22‐item measure assessing four aspects of ED psychopathology, averaged to create a Global score (0–6), with higher scores indicating greater ED psychopathology. The measure has been shown to have strong internal consistency (*α* = 0.95) (Kelly et al. 2013). EDE‐Q was administered at session 1 and 4, and a composite change score was created by subtracting session 1 Global scores from session 4 scores. All analyses using change in EDE‐Q controlled for baseline EDE‐Q.

#### 3.1.3 Procedure

Clinicians provided service users with a link to complete the pre‐session questionnaires online through Qualtrics XM (2005). At their initial assessment with SEDS, service users were assigned a unique participant identification number (PIN) used to identify themselves when completing questionnaires. Records of the assigned PINs were kept on a password‐protected server. Questionnaires incorporated a consent form, including consent for data to be used as part of research. No data were recorded from participants who did not provide full consent. All available questionnaire data collected between January 2022 and August 2023 were exported for analysis.

#### 3.1.4 Data Analysis

All available outcome data were cleaned. Data were included in the analysis only if they could be deanonymized using the PIN provided, enabling additional demographic information to be added from patient records.

Binomial logistic regressions were conducted to identify characteristics associated with ending treatment early. Pearson correlation coefficients explored bivariate associations between variables. To test the overall efficacy of treatment, the percentages of patients meeting the criteria for the Reliable Change Index (RCI; reduction in Global EDE‐Q of ≥ 1.38; Jacobson and Truax 1991) and Clinically Significant Change (CSC; reduction in Global EDE‐Q of ≥ 1.70; Jacobson and Truax 1991) were calculated (Moore, Hinde, and Waller 2021).

Relationships between the five predictor variables (diagnosis type, wait time, baseline depression, baseline anxiety, and therapeutic alliance) and the outcome variable (change in EDE‐Q scores between sessions 1 and 4) were examined using a series of robust multiple regression analyses. Each analysis examined the effect of one predictor variable and three covariates (age, gender, and baseline EDE‐Q) on change in EDE‐Q scores.

Tests to inspect assumptions of normality, linearity, and homoscedasticity were completed for all multiple regression models. Examination of residual scatterplots revealed no issues with normality. No cases in any of the models had a Cook's distance of greater than one and therefore none had an undue influence. However, there was some evidence of nonlinearity and heteroscedasticity, therefore, the decision was made to run robust multiple regressions.

All results from Phase 2 of the study have been reproduced by an independent statistician from the University of Sussex, and their report can be viewed here: https://osf.io/q8bpc.

#### 3.1.5 Missing Data

**FIGURE 1:**
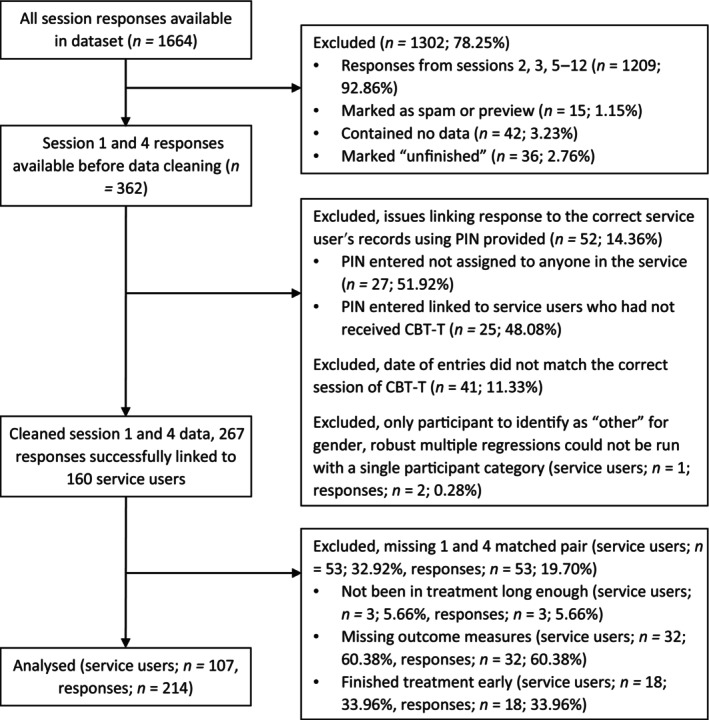
Data cleaning process.

Following cleaning, data were available for 160 service users (Figure 1). Fifty participants were excluded from analysis as they did not have completed outcome measure data for both session 1 and 4. This was due to either missing data (e.g., not completed or entered, *n* = 32), participants finishing treatment prior to session 4 (*n* = 18), or participants not having yet completed session 4 (*n* = 3). One hundred and seven participants could be included in the analyzed sample. Binomial logistic regressions found that participants who were excluded due to not having paired session 1 and 4 data were more likely to be male (Table S2).

## 4 Results

### 4.1 Sample and Preliminary Analysis

Of the total 160 service users, 26 ended treatment before session 5 (16.25%). Binomial logistic regressions found that early finishers were more likely to have higher depression and anxiety at baseline (Table S3). Of the 107 participants included in the analyzed sample, 92.52% of the were female, and the mean age was 32.55 years. 57.94% had a “Binge Eating” type diagnosis and 42.06% had a “Restrictive” diagnosis. Participant characteristics are described in depth in Table 3.

**TABLE 3:** Demographic characteristics of participants in Phase 2.

	Analytic group (*n* = 107)	
Categorical	*n*	%
Gender		
Female	99	92.52
Male	8	7.48
Diagnosis group		
Binge eating	62	57.94
Restrictive	45	42.06
Diagnosis		
Binge‐eating disorder (BED)	32	29.91
Bulimia nervosa (BN)	27	25.23
OSFED—atypical anorexia nervosa (OSFED‐AAN)	36	33.64
OSFED—bulimia nervosa (OSFED‐BN)	3	2.80
OSFED—purging disorder (OSFED‐Purging)	4	3.74
Unspecified feeding or eating disorder (UFED)	5	4.67

Continuous	*M* (SD)	Min–Max
Age (years)	32.55 (11.64)	18–64
BMI	29.84 (9.80)	17.6–56.0

Abbreviations: *M* = Mean; Max = maximum; Min = minimum; SD = standard deviation.

Pearson correlation coefficients between the variables were explored (Table 4). Baseline anxiety and depression, EDE‐Q scores for session 1 and 4, and change in EDE‐Q scores were all significantly correlated with each other. Large significant correlations were found between session 1 and 4 EDE‐Q scores and change in EDE‐Q scores, and between baseline depression and anxiety.

**TABLE 4:** Descriptive statistics and correlations for study variables.

Variable	*M*	SD	Min–Max	Diagnosis type^a^	Depression	Anxiety	Therapeutic alliance	Wait time	Session 1 global EDE‐Q	Session 4 global EDE‐Q	Change in global EDE‐Q
Diagnosis type^a^	0.42	0.50	0–1	—							
Baseline depression	13.60	5.90	3–26	−0.08	—						
Baseline anxiety	11.33	5.95	0–21	0.05	0.69**	—					
Therapeutic alliance	4.13	0.69	2–5	0.10	−0.10	−0.04	—				
Wait time	81.26	34.67	2.29–175.00	−0.28*	0.07	−0.07	−0.02	—			
Session 1 global EDE‐Q	3.59	1.25	0.11–5.75	−0.12	0.44**	0.40**	−0.16	0.05	—		
Session 4 global EDE‐Q	2.97	1.24	0.54–5.75	−0.09	0.39**	0.38**	−0.20	0.18	0.73**	—	
Change in global EDE‐Q	−0.62	0.91	−2.86–2.46	0.04	−0.08	−0.02	−0.05	0.16	−0.38**	0.35**	—

Abbreviations: EDE‐Q = Eating Disorders Examination Questionnaire; *M* = Mean; Max = maximum; Min = minimum; SD = standard deviation.

^a^ 0 = binge eating and 1 = restrictive.

* *p* < 0.01.

** *p* < 0.001.

A paired *t‐*test was conducted to explore change in EDE‐Q scores between sessions 1 and 4. Overall mean scores were significantly lower at session 4 than session 1 (*M*_diff_ = 0.62, 95% CI [0.44, 0.79]), indicating that EDE‐Q scores significantly reduced early in CBT‐T (*t* (106) = 7.04, *p* < 0.001). Less than 1% of patients experienced a clinically significant increase in EDE‐Q scores in the first four sessions. At session 1, 26.17% of service users' EDE‐Q scores were below the cut‐off for remission (2.77; Turner et al. 2015), rising to 43.93% by session 4. After the first 4 sessions, 21.5% of service users met the criterion for the RCI, and 8.41% met the criterion for CSC.

### 4.2 Predictors of Early Change

Robust multiple regression models revealed no significant associations between any of the five predictor variables (diagnosis type, wait time, baseline depression, baseline anxiety, and therapeutic alliance) and change in EDE‐Q scores between sessions 1 and 4 (Table 5). All models accounted for less than 2% of the variation in change in EDE‐Q scores, and the effect sizes were small to medium. Post hoc power analyses were conducted using G*Power (Faul et al. 2009) to evaluate the power of the robust multiple regression model. The achieved power of the five predictors ranged between 0.692 and 0.848, with only wait time having power of over the commonly accepted threshold of 0.80, based on a total sample size of 107 participants and an alpha level of 0.05.

**TABLE 5:** Robust multiple regression models exploring predictors of change in EDE‐Q scores.

Predictor	Estimate	*R* ^2^	SE	95% CI	*t*	*f* ^ 2^	*p*
Unadjusted							
Baseline depression	−0.01	0.003	0.02	−0.04, 0.01	−0.48	0.003	0.631
Restrictive diagnosis	0.10	0.002	0.19	−0.29, 0.44	0.52	0.002	0.606
Therapeutic alliance	−0.23	0.023	0.21	−0.42, 0.28	−1.08	0.023	0.281
Baseline anxiety	−0.01	0.002	0.02	−0.03, 0.02	−0.41	0.002	0.681
Wait time	0.00	0.020	0.00	0.00, 0.01	1.51	0.020	0.135
Adjusted*							
Baseline depression	0.01	0.098	0.02	−0.01, 0.05	0.63	0.108	0.528
Restrictive diagnosis	−0.02	0.092	0.23	−0.35, 0.32	−0.09	0.102	0.925
Therapeutic alliance	−0.28	0.105	0.20	−0.51, 0.17	−1.42	0.117	0.158
Baseline anxiety	0.03	0.112	0.02	−0.01, 0.06	1.37	0.126	0.175
Wait time	0.01	0.124	0.00	0.00, 0.01	1.64	0.141	0.105

Abbreviations: CI = confidence interval; *EDE‐*Q = Eating Disorders Examination Questionnaire; SE = standard error.

* Models adjusted for session 1 EDE‐Q, age, and gender.

## 5 Discussion

This study supports CBT‐T as a potentially beneficial treatment for nonunderweight EDs in clinical settings. Significant decreases in ED psychopathology were demonstrated within four sessions. By session 4, 17.76% of individuals had moved below the clinical cut‐off for remission and 21.5% met the criterion for the RCI.

Qualitative analysis highlighted a number of factors, which clinicians felt were predictive of early change in EDE‐Q. However, quantitative analysis of the five testable variables—therapeutic alliance, wait time, diagnosis, and baseline anxiety and depression—found that none significantly predicted early change. These findings suggest that clinicians' perceptions of the factors which influence early change may not be valid. Previous research has demonstrated that clinicians are typically poor at predicting which patients will do well in treatment, and that clinician judgment is less accurate than statistical methods at predicting outcome (Ægisdóttir et al. 2006; Grove et al. 2000; Meehl 1954). This poor accuracy may, in part, be due to errors in clinical judgment, heuristics including representativeness and availability, and a lack of feedback on the accuracy of their judgments (Grove et al. 2000). In particular, interviews revealed disparities between the emphasis placed by clinicians on therapeutic alliance, compared with the CBT‐T manual. Clinicians felt a strong therapeutic alliance was important for supporting early change. However, quantitative analysis found no relationship, in line with earlier findings (Waller et al. 2018), reflecting a tendency for clinicians to overemphasize the role of therapeutic alliance. The manual does not target the development of alliance (Waller et al. 2019), citing evidence that clinicians overvalue its significance in subsequent symptom change (Brown, Mountford, and Waller 2013, 2014). In ED treatment for adults, it seems early therapeutic alliance is not associated with symptom change; however, early symptom change predicts later therapeutic alliance, irrespective of treatment type, age, or diagnosis (Graves et al. 2017). While the therapeutic relationship is consistently shown to be important in patients' experience of treatment (Graves et al. 2017), clinicians must ensure they do not unintentionally limit patients' outcomes (Waller and Turner 2016) by focusing on therapeutic alliance at the cost of pushing for symptom change (Brown, Mountford, and Waller 2014).

Clinicians felt CBT‐T was less suited to restrictive diagnoses, but quantitative analysis found no relationship between diagnosis type and early change. Clinicians described “adapting” treatment to make it more suitable for restrictive diagnoses, potentially mitigating poorer outcomes. Further research is required to compare differences in outcomes based on diagnosis. To our knowledge, only one other study has attempted this (Keegan and Wade 2023) and most previous CBT‐T research focuses on BN and BED (Keegan, Waller, and Wade 2022). Importantly, it would be beneficial for clinicians to capture and disseminate the adaptations of CBT‐T delivered for restrictive patient groups, enabling formal testing of their efficacy and, ultimately, the possibility of opening new treatment pathways for this population.

Clinicians considered long wait times for treatment detrimental to making early change, as presentation and self‐efficacy may deteriorate while waiting, and patients may feel less prepared and clear on what treatment involves. However, quantitative exploration found no significant relationship between early change and wait time. This contradicts previous ED research showing longer waits are associated with poorer CBT outcomes (Sánchez‐Ortiz et al. 2011), poorer engagement (Bell and Newns 2004), and increased dropout (Carter et al. 2012). Despite our null finding, decreasing wait times for ED treatment is still a pressing concern, and CBT‐T has real‐world value for tackling NHS waiting lists due to its time‐limited nature and potential to be delivered by unqualified staff populations less affected by the healthcare recruitment crisis.

Baseline depression and anxiety did not significantly predict early change in EDE‐Q scores. However, consistent with previous research, high depression and anxiety scores were associated with higher EDE‐Q scores at session 1 (Elran‐Barak and Goldschmidt 2021; Smith et al. 2018). The current study suggests that, while psychological comorbidities may increase risks of leaving treatment early, they are unrelated to outcomes in those that continue. Previous research exploring the influence of depression and anxiety on change in ED psychopathology is inconclusive. Comorbidities have been associated with poorer early change in AN (Le Grange et al. 2014) but not BED (Yurkow, Ivezaj, and Grilo 2023), and their impact on end‐of‐treatment outcomes in CBT‐T remains unclear (Pellizzer, Waller, and Wade 2018; Waller et al. 2018). Further research clarifying the impact of psychological comorbidity on early change in CBT‐T is required to assist clinical decision‐making around treatment allocation.

Several indicators from the interviews could not be tested quantitatively as the relevant data are not routinely collected in clinic. High motivation, readiness to change, self‐efficacy, and commitment to change were all highlighted in our qualitative work as useful indicators of improved clinical outcomes, and these are supported by empirical research findings (Sansfaçon et al. 2020; Vall and Wade 2015; Steele, Bergin, and Wade 2011). Replication of previous findings in the context of clinical care would be a valuable addition to the literature. A further consideration raised by clinicians is the role of trauma. A recent systematic review found that although exposure to trauma was associated with greater dropout in ED treatment, it did not predict outcomes (Convertino and Mendoza 2023). As most individuals with EDs have a trauma history (Brewerton 2019), future CBT‐T research should explore how trauma may impact outcomes, in order to increase understanding of the clinical implications.

### 5.1 Strengths, Limitations, and Directions for Future Research

As the first study exploring predictors of early change in CBT‐T, this research provides a meaningful addition to the literature on psychological treatments for EDs. In addition to representing one of the largest sample sizes to‐date, the study benefited from two key strengths. First, it has high ecological validity from utilizing data drawn from CBT‐T provision in an NHS ED service, meaning the sample was highly representative of the target population, increasing the generalizability of findings. Second, it used an exploratory sequential mixed‐methods design—a particularly beneficial approach given the lack of research in this area. Qualitative interviews provided the first representation of clinical experience in routine CBT‐T delivery, allowing identification of potential predictors of early change and informing subsequent quantitative analyses conducted to test the highlighted factors. Qualitative investigation facilitated deeper analysis, providing detail and possible explanations for findings, and enabling contextualization of quantitative results.

Despite the study's strengths, there were limitations. First, following data cleaning, 33% of the sample was excluded from analysis as they did not have completed measures for both session 1 and 4, with comparison showing that men were more likely than women to be excluded on this basis. Although no significant differences were found for diagnosis, age, BMI, or baseline depression and anxiety, differences could not be determined for session 4 variables including therapeutic alliance and change in EDE‐Q scores. Therefore, it is possible that the pattern of missing responses was not random, potentially impacting findings. Second, due to the nature of the data used, the quantitative predictors were not an exact match for the factors highlighted by clinicians in Phase 1, and many themes from the qualitative findings could not be tested quantitatively. Future studies should aim to quantitatively explore the factors highlighted by clinicians more closely. Additionally, while out of scope for the current analysis, future work could attempt to replicate these findings across a different measurement type, such as the ED‐15 (Tatham et al. 2015), which is completed weekly as part of CBT‐T protocol. Third, the lack of preregistration and sample size calculations are a limitation, and as power to detect significant effects fell just below the threshold of 0.80 for all but one of the predictor variables, the quantitative analysis may have lacked sufficient power. Fourth, clinicians' perspectives appeared highly homogenous. This may reflect consistency in their experiences, strengthening the emergent themes. However, it is possible that the homogeneity may not have occurred organically and was in part shaped by discussions during regular group supervision in which clinicians consider patients' suitability for CBT‐T and factors impacting progress. Finally, patients' and therapists' perspectives on what influences treatment outcomes may differ (Kramer et al. 2008; Westmacott et al. 2010). Future studies should expand on the present findings by interviewing patients, as well as exploring clinician perspectives in other services to help determine the transferability of findings.

## 6 Conclusion

While CBT‐T appears effective for a large proportion of service users, the findings indicate that, like other psychological treatments, it is not suitable for all. This emphasizes the importance of reviewing treatment suitability at session 4, allowing consideration of new care pathways for those who have not made change, and mitigating potential harms from continuing in a therapy misaligned to patient needs. Overall, these findings are of value in providing justification and guidance for further larger‐scale treatment studies, aimed at clarifying the mechanisms determining early change in CBT‐T.

## Author Contributions

**Dana Gatley:** conceptualization, data curation, formal analysis, investigation, methodology, project administration, writing – original draft, writing – review and editing. **Verity Millar‐Sarahs:** data curation, formal analysis, investigation. **Faith Matcham:** conceptualization, methodology, supervision, writing – review and editing. **Amy Brown:** conceptualization, methodology, resources, supervision, writing – review and editing. **Cat Papastavrou Brooks:** conceptualization, methodology, resources, supervision, writing – review and editing.

## Ethics Statement

The study received ethical approval from the University of Sussex Science and Technology C‐REC (ER/DPG21/1) and was registered as a quality improvement project by the Sussex Partnership NHS Foundation Trust (SPFT) Quality Improvement Team on 11th November 2022. NHS ethics approval was not required. All participants provided full consent. The research complied with British Psychological Society guidelines and NHS trust policy for data protection and handling.

## Conflicts of Interest

The authors declare no conflicts of interest.

### Open Research Badges

This article has earned Open Data and Open Materials badges. Data and materials are available at https://osf.io/952rn/.

## Supporting information


**Data S1.** Supporting Information.

## Data Availability

Interview data cannot be publicly shared due to confidentiality. All other data and materials necessary to reproduce the findings reported in this manuscript are available at https://osf.io/952rn/.
