# Supplementary material for: Understanding Early Treatment Response in Brief CBT for Nonunderweight Eating Disorders: A Mixed Methods Study
Source: Int J Eat Disord. 2024 Dec 13;58(3):518–30. doi: 10.1002/eat.24350 (PMC11891623; doi:10.1002/eat.24350)
Supplement: Supplementary file 1 — Data S1. Supporting Information. [file EAT-58-518-s001.docx]

**Supplementary Material**

**Supplementary Data 1: Clinician Semi-Structured Interview Topic Guides**

*Semi-structured interview topic guides used during interviews with clinicians, including 7 questions and accompanying prompts.*

**Interviewer:**

“Thank you for taking part in this interview. I’m going to be asking you some questions about

your experience of CBT-T, so that we can develop a better understanding of clinicians’

experiences and thoughts on this treatment. This interview will be recorded, and will take no

more than an hour. You can let me know if you would like to take a break at any point, and

you are also free to end the interview at any time if you’d like to do so. Do you have any

questions before we start?”

**Questions**

1. What is your involvement with CBT-T within the service?

*Prompts:*

- *What is your role in the service?*
- *How long have you delivered CBT-T for?*
- *How many patients do you have on your caseload for CBT-T?*
- *Do you deliver other treatments? Or just CBT-T?*

1. What has your experience been of delivering CBT-T?

*Prompts:*

- *How do you find delivering CBT-T to service users?*
- *What sorts of patients do you see? What are the criteria for receiving CBT-T treatment? BMI? Diagnosis?*
- *Do you enjoy it? Find it challenging/difficult?*
- *What are the challenges of delivering CBT-T?*
- *How does your experience of delivering CBT-T compare to other therapies?*

1. How do you think service users experience CBT-T?

*Prompts:*

- *Do you think service users like CBT-T?*
- *Do you think CBT-T is an effective treatment for service users?*
- *Is it effective for all patient? What patients is it not effective for? Why?*
- *What do you think service users find difficult about CBT-T? What do you think they find helpful? Why do you think they found these aspects helpful/difficult?*
- *How do you think CBT-T compares with other therapies?*
- *Do you think it has specific benefits? Weakness/challenges? In comparison to other therapies*
- *How do they find self-monitoring?*

1. So I know CBT-T stresses the importance of early change, could you please tell me a little bit about this?

*Prompts:*

- *Why does CBT-T stress early change? What is the importance of early change?*
- *Do you inform patients of the need for early change prior to treatment?*
- *What happens at session 4?*
- *Do patients respond well to the push for early change?*
- *What do you think encourages/supports early change in CBT-T? Motivation?*
- *Support systems? Place in recovery journey?*
- *How are the goals for changes each week created? Service-user involvement?*
- *What makes early change difficult/limits early change?*
- *How do you support people with the anxiety that making changes causes?*
- *Are there any factors/characteristics that predict early change (or lack of change)?*
- *Is motivation important for early change? Do you measure/develop it?*

1. What do think causes drop-out/non-completion of treatment in CBT-T?

*Prompts:*

- *How many of your patients do not complete the 10 sessions?*
- *Why do patients not complete CBT-T?*
- *What makes a patient likely to drop-out? Are there specific characteristics?*
- *Are there any factors or characteristics that you think predict drop-out/noncompletion? Motivation? Life circumstances? Support systems?*
- *Is attrition caused more by service users dropping-out/not attending or by therapist suggestion at session 4?*
- *What are your thoughts on ending treatment at Session 4 if the patient has failed to make changes? How does this make you feel emotionally? How do you think patients experience this? Why do you think this is part of the manual?*
- *What do you think could help to reduce drop out?*

1. Please can you talk to me about the role of the therapeutic relationship in CBT-T?

*Prompts:*

- *What effect do you think the therapeutic relationship has?*
- *Is it important? Why? Important for change?*
- *How do you experience the therapeutic relationship?*
- *How do you think patients experience the therapeutic relationship?*
- *Do you find any aspect of the therapeutic relationship difficult?*
- *What is the therapeutic relationship in the context of pushing for early change?*

1. Do you have any thoughts or suggestions on how CBT-T could be improved?

*Prompts:*

- *For service users? For clinicians?*
- *What support do you have in place for CBT-T? What support would be useful?*
- *Would you change anything about CBT-T and how you have to deliver it?*
- *Is there anything else that you would like to mention*

| **Supplementary Table 1**  *Summary of themes from thematic analysis of interviews, and example quotes for each code* | |
| --- | --- |
| **Theme** | **Example Quotes** |
| **Service-user Factors** |  |
| 1. **Attitudes to making change** | |
| Motivation and readiness to change | *… the people who are not very attached to their eating disorder and are really motivated and they say, I'm 10 out of 10 motivated. I'm ready to make change right now […] They're not contemplating change. They would do it right now if someone told them, if someone just told them what to do, […] Those people will do really well.* (Clinician 2) |
| Role of eating disorder in identity | *The patients who have, yeah, those sort of eating disorders where it's really significant to them and something that they, they want to hold onto. I don't feel like it's probably the most suitable treatment just cause it doesn't address what needs to be, um, given a lot of time.* (Clinician 6) |
| Open to trying changes despite anxiety | *… it's okay to be scared, like of things like weighing yourself, of things like doing food diaries or eating fear foods or eating regularly, but the people that do the best are the people that are scared and kind of can do it anyway because they understand why we're, like they understand the rationale, and they're not too, like resistant to that.* (Clinician 2) |
| Self-efficacy | *I think, so people I work with, um, who've sort of believed that they're not able to make changes […] That can create a barrier as well, if someone doesn't feel that it's possible to make that change, that can make things more difficult.* (Clinician 7) |
| Commitment to doing the 'work' | *I think as a clinician you do get a sense of who's gonna do well in CBT-T, and when you get the right patient who's willing to do the homework, willing to do all of the stuff that you need to do outside of the therapy session, it goes really well and it has got like excellent results …* (Clinician 3) |
| Benefits of ‘diving’ immediately into changes | *I think if they're making kind of really gradual changes, I think if potentially she only did breakfast and actually her weight didn't jump, she's still gonna have that worry that, okay, if I have lunch and dinner, my weight could jump. So I think it's almost like throwing herself in the deep end, seeing what happens and actually like facing kind of their biggest fear kind of front on.* (Clinician 1) |
| 1. **Diagnosis-related factors** | |
| Effective for binge-eating diagnoses | *… binge-eating disorder and bulimia nervosa seem to do particularly well with CBT-T, and that's sort of the OSFED variants of, of those conditions as well.* (Clinician 7) |
| **Theme and Codes** | **Example Quotes** |
| Considerations for implementation for restrictive diagnoses | *I don't think it necessarily works as well for kind of OSFED or atypical AN. I think there's a lot of things in there, even kind of the talk of binging. Some patients will be put through and they're not binging at all. Um, and I think then trying to adapt to sessions can be quite difficult.* (Clinician 1) |
| Duration of eating disorder | *… how long they've had their eating disorder for, might hinder change. Um, so you said, you know, it's, it's so ingrained, like, again, thinking about your own habits that you have and you've done it your whole life, then to suddenly change it, I think that'd be quite difficult.* (Clinician 5) |
| 1. **External mitigating circumstances** | |
| Importance of social support | *… for patients that have like either a supportive partner or friends or family around them, like they're sometimes more likely to make those early changes. I think it’s kind of having somebody else to kind of keep them accountable as well.* (Clinician 1)  *… social support is so incredibly important for any type of therapy and recovery …* (Clinician 8) |
| Life events can limit engagement | *… the timing of it I think is really important. You know, what else they've got going on in their life. If they're like moving house, if they've suffered a bereavement, for example, like it's not really gonna be, yeah, let's, let's do some work on your eating disorder.* (Clinician 5) |
| Comorbidity | *… if someone, alongside eating disorder is struggling with, um, anxiety […] the idea of making changes in one area, you know, they can voice a lot of thoughts and, um, sort of catastrophisation around the idea, which can hinder their, their ability to, to make those changes […] if someone's very low in mood or struggling with depression, then um, that can also impact, um, motivation and ability to do things […] So actually the idea of eating regularly, um, is difficult, but not necessarily because the eating itself, but actually the doing things …* (Clinician 7) |
| Trauma history | *… if they, they, they haven't got, […] some other coping skills which you can build on, then, um, perhaps they might drop out. Um, and that's normally linked to those like underlying traumas that maybe haven't been resolved or dealt with […] So they're dealing with a lot and it's not something that can be fully covered within the scope of the CBT-T.* (Clinician 3) |
| **Therapist Factors** |  |
| 1. **Significance of the therapeutic alliance** | |
| Disagreement with manual’s stance | *The manual has this like philosophy about how it doesn't really matter about the therapeutic relationship and, um, basically, you know, it says, oh, you don't, you don't have to like me, kind of thing, like as a, as a way of doing it. But in my experience that doesn't, doesn't really work that well.* (Clinician 3) |
|  |  |
| **Theme and Codes** | **Example Quotes** |
| Supports early change | *… if someone feels more comfortable opening up to you or is exploring things, then I think they could benefit from that. You know, if there's something that's going on, um, and they're not able to express it, then that, they could be things that are impacting therapy or their ability to make changes that you just don't know about […] you might not be able to do anything, you know, depending on the nature of it, but just letting it, putting it out there and both knowing where the person stands can be helpful.* (Clinician 7) |
| Increases motivation | *… the patients that I've had kind of, or I feel like I've had a better therapeutic relationship with, um, I think that in itself kind of almost fosters a bit of motivation for them […], especially if the patients that haven't had necessary people in their life that are kind of like motivating them, keeping them accountable, […] I think then if they have this therapeutic relationship in CBT[-T], they're more like, okay, actually someone thinks I can kind of, obviously you don't say you, you can do it, but kind of they know you're on kind of their side …* (Clinician 1) |
| Builds trust | *I think if you don't have that rapport, you know, you don't work on building that therapeutic relationship and sort of, I suppose, developing that trust in the person you're working with that actually you've only just met, […] Asking someone to make changes, asking someone to trust you and to sort of believe what you're telling them and to have, have faith, have trust that if they do these things, it could help and could, because different things work for different people so sometimes it is about trial and error. And I do say that said, you know, we can try this see how it works for you then. And I don't, I think that would just be detrimental if you didn't have that rapport.* (Clinician 7) |
| 1. **Therapist confidence** | |
| Training and experience | *… [if] they don't believe in the, in the therapy that they're having. They don't believe in, in the person delivering it let's say, any of these factors, um, that, that leads to a reduced efficacy in the treatment. I think. Um, um, so one of the things that determines that, I think, is therapist confidence, and confidence comes from experience and knowledge. So I think you have to be quite versed in the therapy and confident in your capability to deliver it …* (Clinician 8) |
| Anxiety about pushing for change | *… the more confident you get with delivering CBT-10, the more comfortable you are with pushing someone, um, the right amount because it's like, because it's a therapy that's so focused on like making active change, you do have to push people quite a lot and you worry if you're pushing them a bit too much and then they might drop out or run away or not want to do it …* (Clinician 2) |
| **Service and CBT-T Factors** |  |
| 1. **Pre-treatment variables** | |
| **Theme and Codes** | **Example Quotes** |
| Impact of waiting list | *… one of the things she said was, you know, like “my expectations weren't set up for this. No one told me I was gonna have to eat three meals”. And I think because she'd been on a waiting list for such a long time, […] and no one told her after assessment like, this* |
|  | *is CBT-10, you're on the waiting list for this. […] And then suddenly she gets a call from me after, I dunno, however long she'd been waiting, probably over a year, going, okay, well we've got, we've got a slot for you in treatment. She has no idea what treatment is …* (Clinician 2)  *… if you could reduce assessment wait time, that would probably help as well, because people have like more self-efficacy when they're first recognizing the issue and they're taking the initiative to get on a wait list. […] bulimia, particularly deteriorates on waiting lists.* (Clinician 8) |
| Pre-treatment therapeutic contact | *… I think having that orientation session is often really helpful cuz then people have come back to me and said, oh, I don't actually know if this is for me before I've started treatment […] it allows people to really think, am I ready to do something quite gung-ho.* (Clinician 4)  *So maybe kind of more like review appointments or check-ins between assessment and the start of CBT[-T] to almost kind of get that motivation and start those kind of small early changes, so then when they start treatment, actually, it's not like a massive surprise and they've already started to kind of get the ball rolling.* (Clinician 1) |
| 1. **CBT-T Format** | |
| Length and pace | *… 10 sessions is just not enough for everybody …* (Clinician 2)  *… I think for some people, […] making all the changes is just, was too overwhelming and the pace was too fast.* (Clinician 6) |
| Structured and manualised | *… it difficult for me because I have like a set protocol that I need to follow, and even if that's not completely what the client needs, I still have to deliver that.* (Clinician 2) |
| 1. **Therapeutic suitability** | |
| Issues with patient suitability | *… CBT[-T] is quite a, like specific treatment for a specific group of people. So I think if there was another treatment option, […] for those people that actually are normally kind of faced with not a lot of motivation or kind of actually they've got other things going on […], I think that would reduce dropout from CBT[-T].*  (Clinician 1) |

**Supplementary Table 2:**

*Logistic regression analysis of the differences in baseline characteristics between service-users included in the analysis and those excluded*

|  | **Analysed Sample (*n* = 107, 66.46% of total)** | | | | |
| --- | --- | --- | --- | --- | --- |
|  | **OR** | **Log Odds** | ***p*** | **95% CI** | |
|  |  |  |  | **Low** | **High** |
| **Gender** |  |  |  |  |  |
| Male | - | - | - | - | - |
| Female | 4.02 | 1.39 | .004* | 0.45 | 2.39 |
| **Diagnosis Group** |  |  |  |  |  |
| Binging | - | - | - | - | - |
| Restrictive | 1.41 | 0.34 | .325 | -0.33 | 1.04 |
| **Diagnosis** |  |  |  |  |  |
| Bulimia Nervosa | - | - | - | - | - |
| Binge Eating Disorder | 0.79 | -0.24 | .605 | -1.14 | 0.65 |
| ARFID | 0.00 | -17.38 | .994 | *NA* | 471.46 |
| OSFED | 0.00 | -17.38 | .994 | *NA* | 471.46 |
| OSFED – Bulimia Nervosa | 0.27 | -1.32 | .102 | -3.04 | 0.23 |
| OSFED – Atypical Anorexia Nervosa | 1.07 | 0.06 | .889 | -0.86 | 0.97 |
| OSFED – Purging Disorder | 1.78 | 0.58 | .623 | -1.46 | 3.60 |
| Unspecified Feeding or Eating Disorder | 6956160.35 | 15.76 | .988 | -95.22 | *NA* |
| Age (years) | 1.01 | 0.01 | .617 | -0.02 | 0.04 |
| BMI | 1.03 | 0.03 | .230 | -0.02 | 0.08 |
| Wait Times (weeks) | 1.00 | 0.00 | .812 | -0.01 | 0.01 |
| Baseline Depression | 1.01 | 0.01 | .665 | -0.04 | 0.06 |
| Baseline Anxiety | 1.01 | 0.01 | .655 | -0.04 | 0.07 |

OR = Odds Ratio; CI = Confidence Intervals

**p < .05. **p < .001.*

*Note:* Complete confidence intervals could not be calculated for specific demographic categories due to small sample sizes within these groups

**Supplementary Table 3:**

*Logistic regression analysis of the differences in baseline characteristics of patients who finished treatment early (before session 5) compared to those who completed the first 5 sessions*

|  | **Finished Treatment Early**  **(*n* = 18, 11.18%)** | | | | |
| --- | --- | --- | --- | --- | --- |
|  | **OR** | **Log odds** | ***p*** | **95% CI** | |
|  |  |  |  | **Low** | **High** |
| **Gender** |  |  |  |  |  |
| Male | - | - | - | - | - |
| Female | 0.76 | -0.27 | .659 | -1.39 | 1.06 |
| **Diagnosis Group** |  |  |  |  |  |
| Binging | - | - | - | - | - |
| Restrictive | 1.43 | 0.36 | .407 | -0.50 | 1.22 |
| **Diagnosis** |  |  |  |  |  |
| Bulimia Nervosa | - | - | - | - | - |
| Binge Eating Disorder | 0.48 | -0.73 | .249 | -2.03 | 0.50 |
| ARFID | 0.00 | -14.14 | .992 | *NA* | 282.26 |
| OSFED | 23853796.13 | 16.99 | .991 | -279.45 | *NA* |
| OSFED – Bulimia Nervosa | 1.66 | 0.51 | .590 | -1.57 | 2.27 |
| OSFED – Atypical Anorexia Nervosa | 0.85 | -0.16 | .776 | -1.29 | 0.99 |
| OSFED – Purging Disorder | 4.14 | 1.42 | .190 | -0.83 | 3.69 |
| Unspecified Feeding or Eating Disorder | 1.04 | 0.04 | .977 | -3.02 | 2.14 |
| Age (years) | 0.97 | -0.03 | .191 | -0.07 | 0.01 |
| BMI | 0.96 | -0.04 | .198 | -0.11 | 0.02 |
| Wait Time (weeks) | 1.00 | 0.00 | .494 | -0.02 | 0.01 |
| Baseline Depression | 1.11 | 0.10 | .004* | 0.04 | 0.18 |
| Baseline Anxiety | 1.09 | 0.09 | .027* | 0.01 | 0.17 |

*Note:* Complete confidence intervals could not be calculated for specific demographic categories due to small sample sizes within these groups

OR = Odds Ratio; CI = Confidence Intervals

**p < .05. **p < .001.*
